# Glial Cell Niche Induces Astrocytic Differentiation of NSCs via the BMP4–Smad1/5/8 Signaling Pathway

**DOI:** 10.3390/ijms27177642

**Published:** 2026-08-26

**Authors:** Meiqi Sun, Simeng Cui, Xinyi Wang, Yali Fu, Xiaoxuan Hu, Ihtisham UI Haq, Faisal Yaqoob, Zixuan Zhang, Ruolan Tan, Wanting Zhao, Hongli You, Jiajing Xia, Jing An, Xinlin Chen, Kaige Ma, Haixia Lu

**Affiliations:** Institute of Neurobiology, School of Basic Medical Sciences, Xi’an Jiaotong University Health Science Center, Xi’an 710061, China; smqmina44@163.com (M.S.); 18192091178@163.com (S.C.); wangxy_95095@163.com (X.W.); f19965482830@163.com (Y.F.); xiaoxuanhu21@163.com (X.H.); ihtishamkhan66@gmail.com (I.U.H.); faisal_yaqoob@outlook.com (F.Y.); zhangzixuan_dsa@163.com (Z.Z.); tanruolan19930117@163.com (R.T.); douding_1216@163.com (W.Z.); youhongli2002@163.com (H.Y.); 17752863755@163.com (J.X.); anjing@mail.xjtu.edu.cn (J.A.); chenxl@mail.xjtu.edu.cn (X.C.)

**Keywords:** glial cell niche, NSCs, astrocytic differentiation: BMP4

## Abstract

Specific niche signals play a vital role in supporting the proliferation and differentiation of neural stem cells (NSCs) throughout neural development and neural regeneration. Glial cells, including astrocytes and microglia, are key components of the stem cell niche. However, their interactions with NSCs remain poorly understood. To explore these interactions, primary NSCs isolated from rat embryos were cultured in either glial cell-derived conditioned medium (CM) or co-cultured with mixed glial cells via Transwell inserts. NSC viability, proliferation, differentiation, and the underlying mechanism involving BMP4 signaling were evaluated. Results showed that microglia and astrocytes were closely localized with NSCs in the subventricular zone (SVZ) of rat brains at postnatal day 14. After being cultured with CM or glial co-culture, NSC viability and astrocytic differentiation were significantly enhanced, while cell proliferation and neuronal differentiation were reduced. BMP4 secretion by both astrocytes and microglia as well as Smad1/5/8 and Stat3 phosphorylation in NSCs was also elevated, indicating that the BMP4–Smad1/5/8 signaling pathway was involved in the effects of the glial cell niche on NSC behaviors. These findings suggest that glial cells regulate NSCs’ fate through BMP4-mediated signaling, providing potential targets for future therapeutic strategies in neurogenesis and regeneration.

## 1. Introduction

Neural stem cells (NSCs) reside in the subventricular zone (SVZ) of the lateral ventricles and the subgranular zone (SGZ) of the hippocampal dentate gyrus throughout the lifespan of mammals [1,2,3]. The specific niche signals, including the cellular components, extracellular matrix, blood vessels and other complicated physicochemical properties in the SGZ and SVZ, are essential for NSC proliferation and differentiation during neural development and regeneration [4,5,6,7]. Both astrocytes and microglia are important cellular components in the NSC niche [4,8].

During neural development and regeneration, astrocytes and microglia are closely localized to NSCs and differentially regulate NSC behaviors in response to diverse environmental stimuli [4,8,9,10,11,12]. Several earlier investigations have revealed that astrocytes induce neuronal differentiation of NSCs in adult mice [2,13]. In the late embryonic phase of the central nervous system (CNS), which refers to the period of astrogliogenesis, NSCs mainly differentiate into astrocytes [8,12]. Similarly, at the site of CNS injury, most grafted NSCs differentiate into astrocytes [14,15,16]. However, particular triggers of signal change remain to be uncovered.

Microglia are non-ectodermal cells. They not only affect neurogenesis and astrogenesis during neural development, but also serve as innate immune cells after brain injury [10,11]. During neural development, microglial progenitors enter the neuroepithelium at a very early stage, embryonic day 10.5 (E10.5) in rodents, prior to neurogenesis, and continuously contact NSCs throughout the neurodevelopment period [10,11]. Microglia are known to regulate neurogenesis and synaptic pruning, and their maturation timeline is synchronized with the progression of neurogenesis [17,18]. In the adult brain, microglia exhibit phagocytic activity and release growth factors or cytokines to support the function of adult neurons [17]. In brain injury and aging, microglia also interact with astrocytes and regulate the inflammatory response and offer the signals for the astrocytic differentiation of NSCs [19].

Available findings confirm that astrocytes and microglia exert influences on neurodevelopment. Nevertheless, little is known about the glial cell niche and the interaction between various glial cells, and the possible effects of this niche on NSC biological behaviors in terms of proliferation and differentiation have not been sufficiently investigated. It has been proved that cytokines and small molecular compounds and proteins are involved in determining the fate of NSCs [20]. Among these factors, bone morphogenetic proteins (BMPs) that are highly expressed during late embryonic development exert essential effects on regulating NSCs’ survival, maturation and differentiation [21,22]. Therefore, it is worth clarifying the potential value of BMP4 within the glial cell niche.

To clarify the regulatory effects of the glial cell niche on NSC behaviors and to identify the specific signaling molecules, in this study the spatial distribution of glial cells and NSCs in the developing rat brain was explored. Subsequently, the in vitro glial cell niche system was established via culturing astrocytes and microglia together. After co-culturing the NSCs with the glial cell niche, via CM or the Transwell system, the viability, proliferation and differentiation of NSCs were then measured. Cytokines within the niche were detected and the effects of BMP4 were further confirmed. The findings will uncover the influence of microglia and astrocytes on NSC behaviors and may provide novel insights for developing new strategies to improve the functional potential of NSCs.

## 2. Results

### 2.1. Microglia/Astrocytes Localized Closely to NSCs in the SVZ of Rat Brains During Postnatal Development

**Figure 1:**
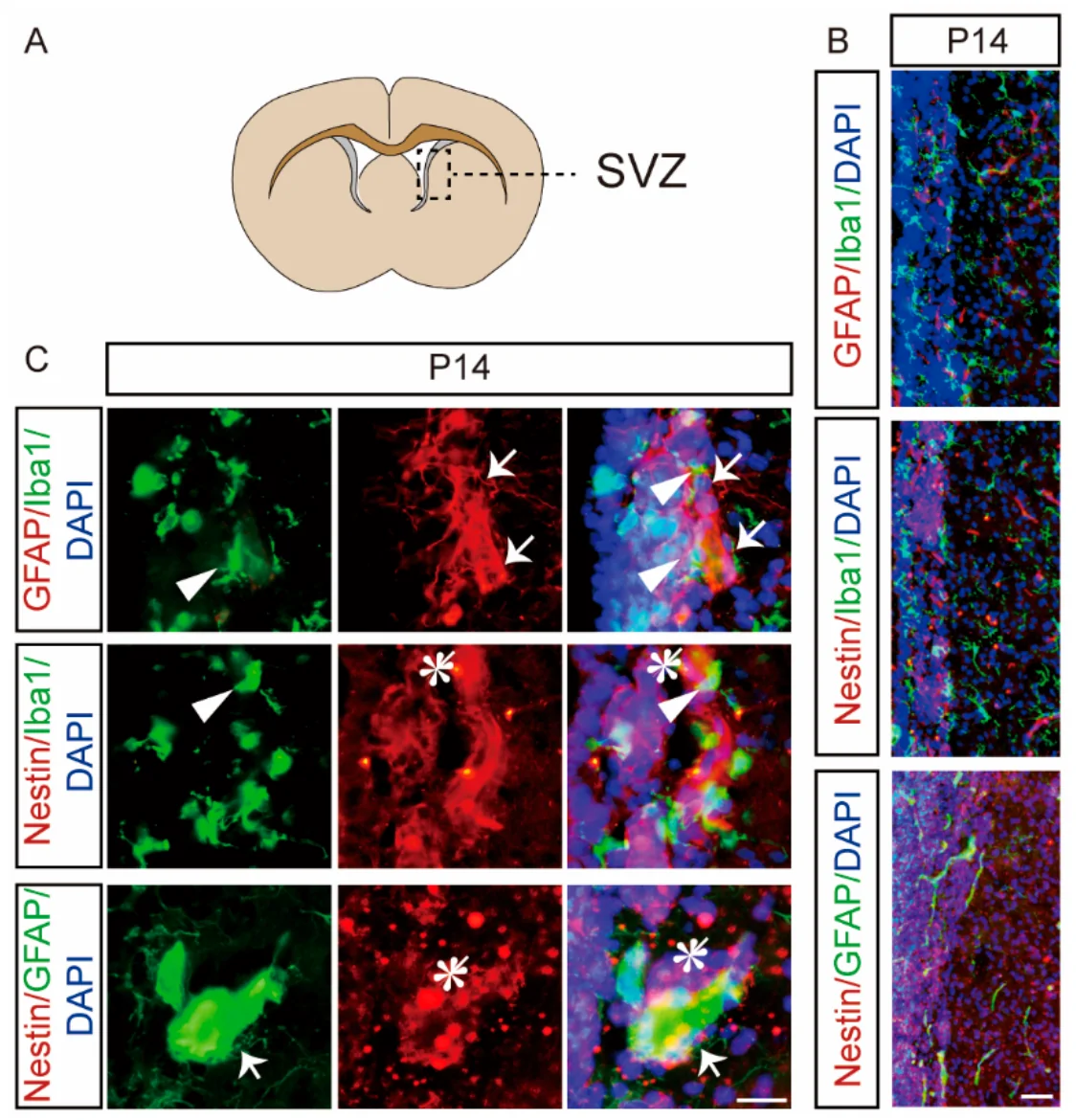
Spatial colocalization of microglia, astrocytes and NSCs in the SVZ of rats at P14. (**A**) Schematic representation of the SVZ region. (**B**) Immunostaining of GFAP-, Iba1- and Nestin-positive cells in the SVZ. (**C**) Enlarged image of the SVZ showing the GFAP-positive astrocytes (arrow), Iba1-positive microglia (arrowhead) and Nestin-positive NSCs (asterisk). Cell nuclei were stained with DAPI (blue). Scale bar = 20 μm.

To explore the potential impact of glial cells on NSCs, the distribution of glial cells in the neurogenic SVZ was examined. The results showed that a large population of Nestin-positive NSCs was observed (Figure 1A–C), and GFAP-positive astrocytes and Iba1-positive microglia were spatially close to NSCs in the SVZ of rats at postnatal day 14 (P14) (Figure 1C).

### 2.2. An In Vitro Glial Cell Niche Was Established by Co-Culturing Microglia and Astrocytes

**Figure 2:**
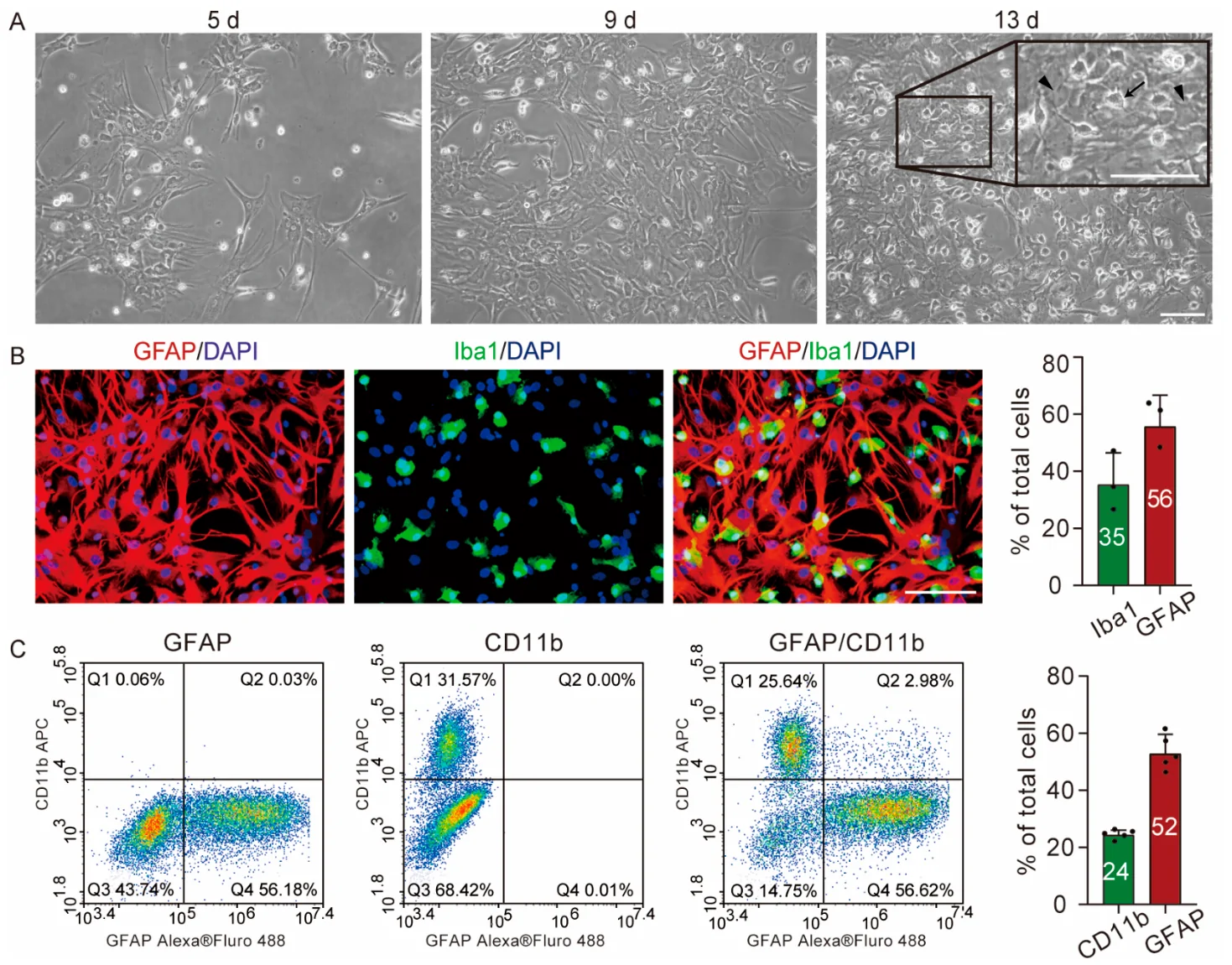
Establishment of a glial cell niche consisting of microglia and astrocytes. (**A**) Glial cell culture images showing astrocytes (arrowhead) tightly adhered to the bottom of culture flasks, whereas microglia (arrow) were distributed above the astrocyte layer, scale bar = 50 μm. (**B**) Immunofluorescence identification and quantification of cultured glial types at day 13. Iba1-labeled (green) microglia and GFAP-labeled (red) astrocytes (*n* = 3). Cell nuclei were stained with DAPI (blue), scale bar = 50 μm. (**C**) Flow cytometric identification and compositional quantification of cultured glial cell types. CD11b (APC)-labeled microglia and GFAP (Alexa Fluor 488)-labeled astrocytes (*n* = 5).

Mixed glial cells were isolated from the cerebral cortex of P1 rats and cultured for 13 days. Within the co-culture system, microglia were in a semi-suspended state, whereas astrocytes firmly adhered to the bottom of the flask (Figure 2A). Quantification of positively stained cells showed that approximately 35% were Iba1-positive microglia and 56% were GFAP-positive astrocytes (Figure 2B). Flow cytometry further indicated that 24% of viable cells were CD11b-positive microglia and 52% were GFAP-positive astrocytes (Figure 2C).

### 2.3. Glial Cell Niche Modulated the Morphology and Viability of NSCs

**Figure 3:**
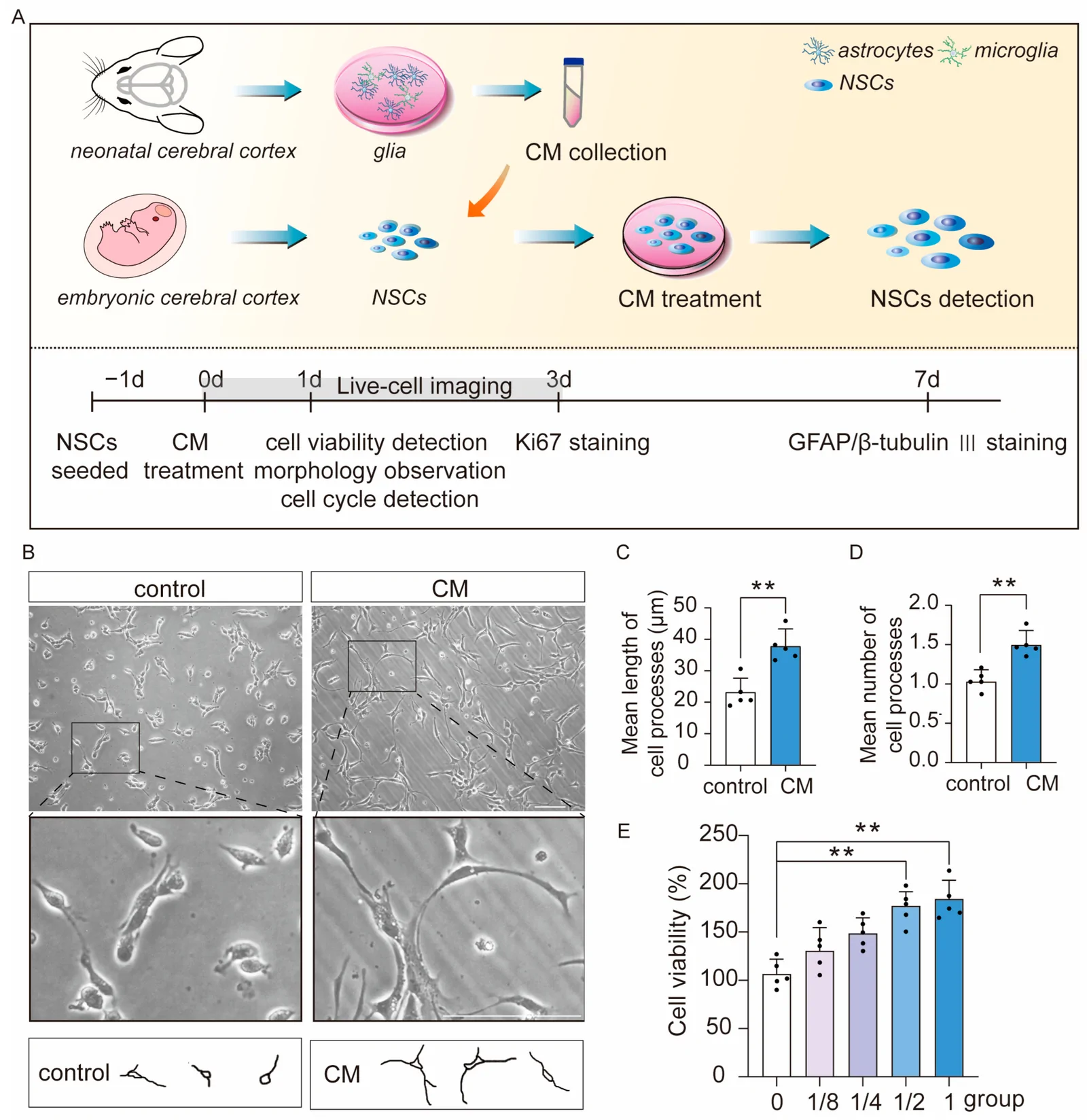
Effects of glial cell niche on NSCs morphology and viability after culture in CM. (**A**) Schematic diagram of the CM collection and NSCs treatment. (**B**) Cell morphology observation under light microscopy at 24 h of culture, scale bar = 50 μm. (**C**,**D**) Quantification of the number and the length of cell processes (*n* = 5). (**E**) NSCs’ viability following treatment with various concentrations (volume ratio) of CM (*n* = 5). Data were analyzed using Student’s *t*-test (**C**,**D**) or one-way ANOVA followed by LSD post hoc test (**E**), ** *p* < 0.01.

CM collected from the mixed glial cell culture system served as the glial cell niche, and NSCs’ morphology and viability were observed (Figure 3A). After 1 day of culture in CM, NSCs attached to the bottom of the flask and displayed different morphologies (Figure 3B). A significant increase was observed in both the number and length of cell processes (Figure 3C,D), indicating the morphological maturation of NSCs. NSCs’ viability was enhanced, particularly when the concentration of CM exceeded 50% (Figure 3E).

### 2.4. Glial Cell Niche Suppressed the Proliferation of NSCs

**Figure 4:**
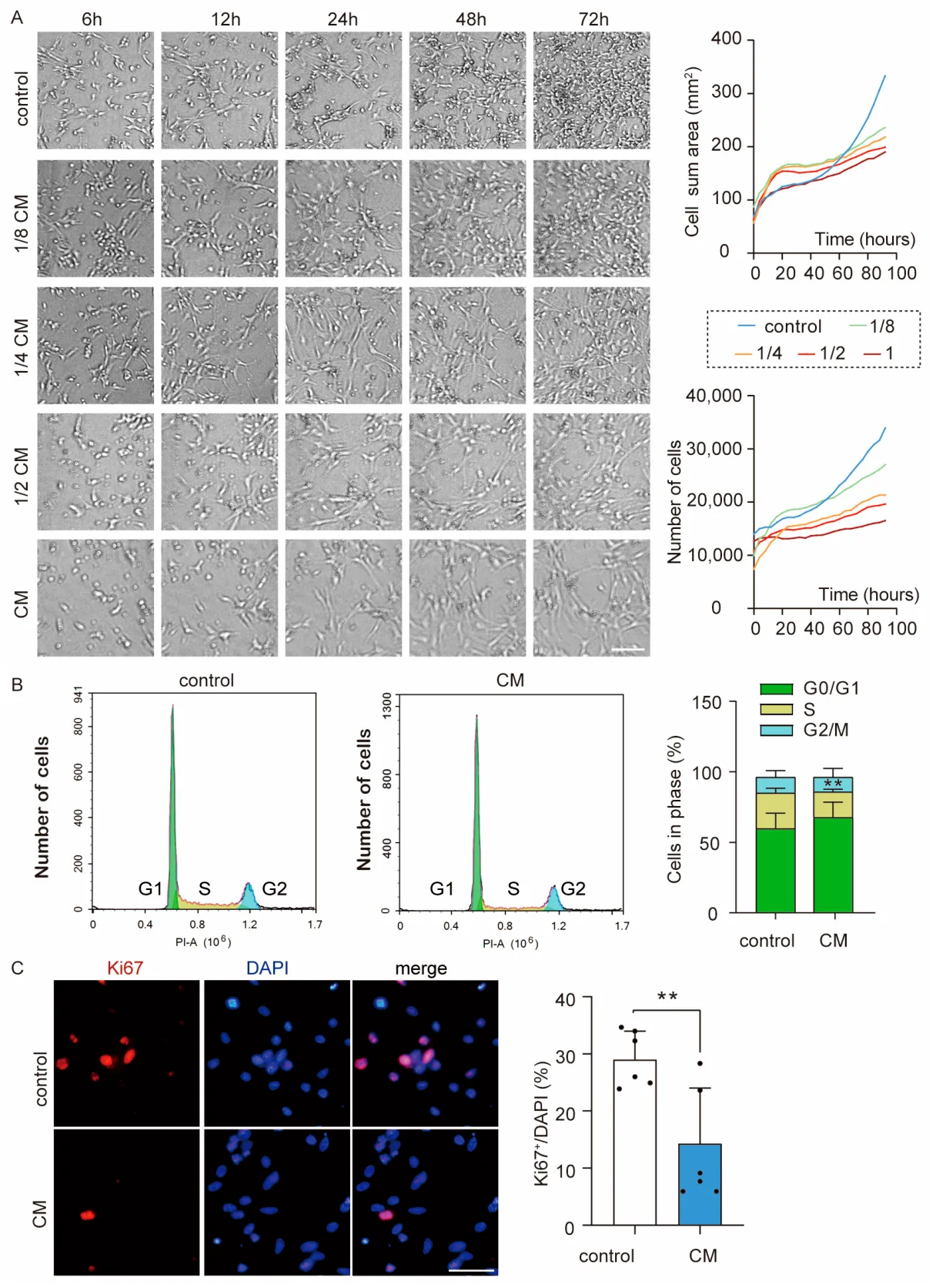
Effects of glial cell niche on NSCs’ proliferation after culture in CM. (**A**) Live-cell imaging of cell growth over a 3-day period, scale bar = 50 μm. (**B**) Quantitative analysis of cell cycle distribution (G0/G1, S, G2/M) using flow cytometry (*n* = 5). (**C**) Immunostaining and quantification of Ki67-positive cells (red) after 3-day treatment (*n* = 6). Cell nuclei were stained with DAPI (blue). Scale bar = 20 μm. Student’s *t*-test, ** *p* < 0.01.

The total number and cell-covered area of NSCs were measured using live-cell imaging. The results indicated that with increasing CM concentration, the speed of cell growth slowed down over the 3-day culture period (Figure 4A), suggesting an inhibitory effect on NSCs’ proliferation. A concentration-dependent reduction in cell-covered area was also observed. After 24 h of culture with CM, flow cytometry revealed a reduced proportion of NSCs in the S phase of the cell cycle (Figure 4B). Consistent with this, immunostaining showed a reduced percentage of Ki67-positive NSCs at day 3 of culture in CM (Figure 4C).

**Figure 5:**
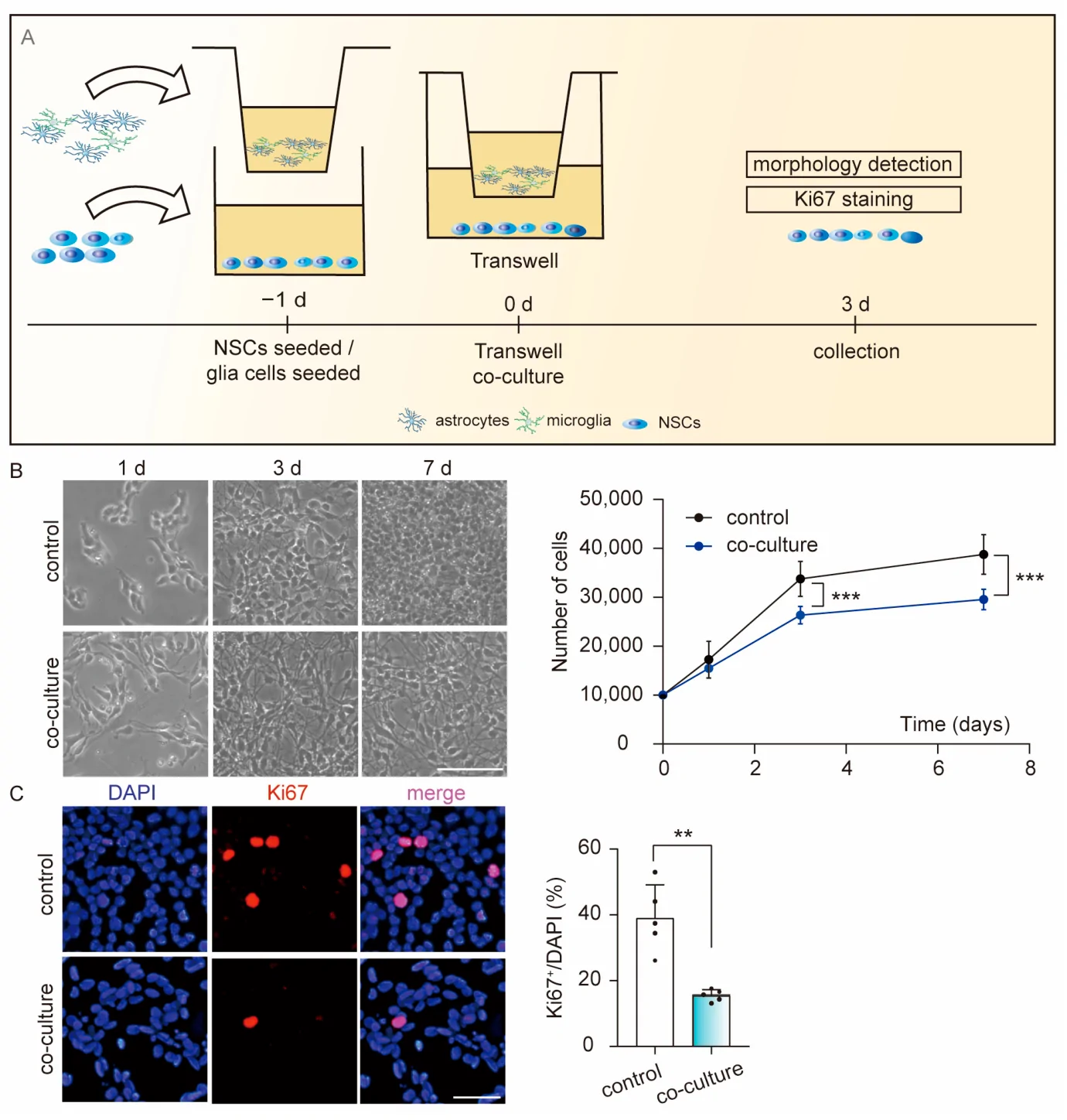
Effects of glial cell niche on NSCs’ proliferation after co-culture in the Transwell system. (**A**) Schematic diagram of the Transwell co-culture system. (**B**) Observation of cell morphology by light microscope and generation of growth curves based on cell counting, scale bar = 50 μm. (**C**) Immunostaining and quantification of Ki67-positive cells (red) after 3-day co-culture (*n* = 5). Cell nuclei were stained with DAPI (blue). Scale bar = 20 μm. Data were analyzed using two-way ANOVA followed by Sidak’s post hoc test (**B**) or Student’s *t*-test (**C**), ** *p* < 0.01, *** *p* < 0.001.

To further confirm the inhibitory effect of the glial cell niche on NSCs’ proliferation, continuous exposure to glial cells was undertaken by co-culturing NSCs with mixed glial cells via Transwell inserts (Figure 5A). According to the growth curve, cell numbers on days 3 and 7 were significantly decreased compared with the control (Figure 5B). Similarly, a significant reduction in cell proliferation, as indicated by the proportion of Ki67-positive cells, was observed on day 3 of co-culture (Figure 5C).

### 2.5. Glial Cell Niche Promoted Astrocytic Differentiation of NSCs

**Figure 6:**
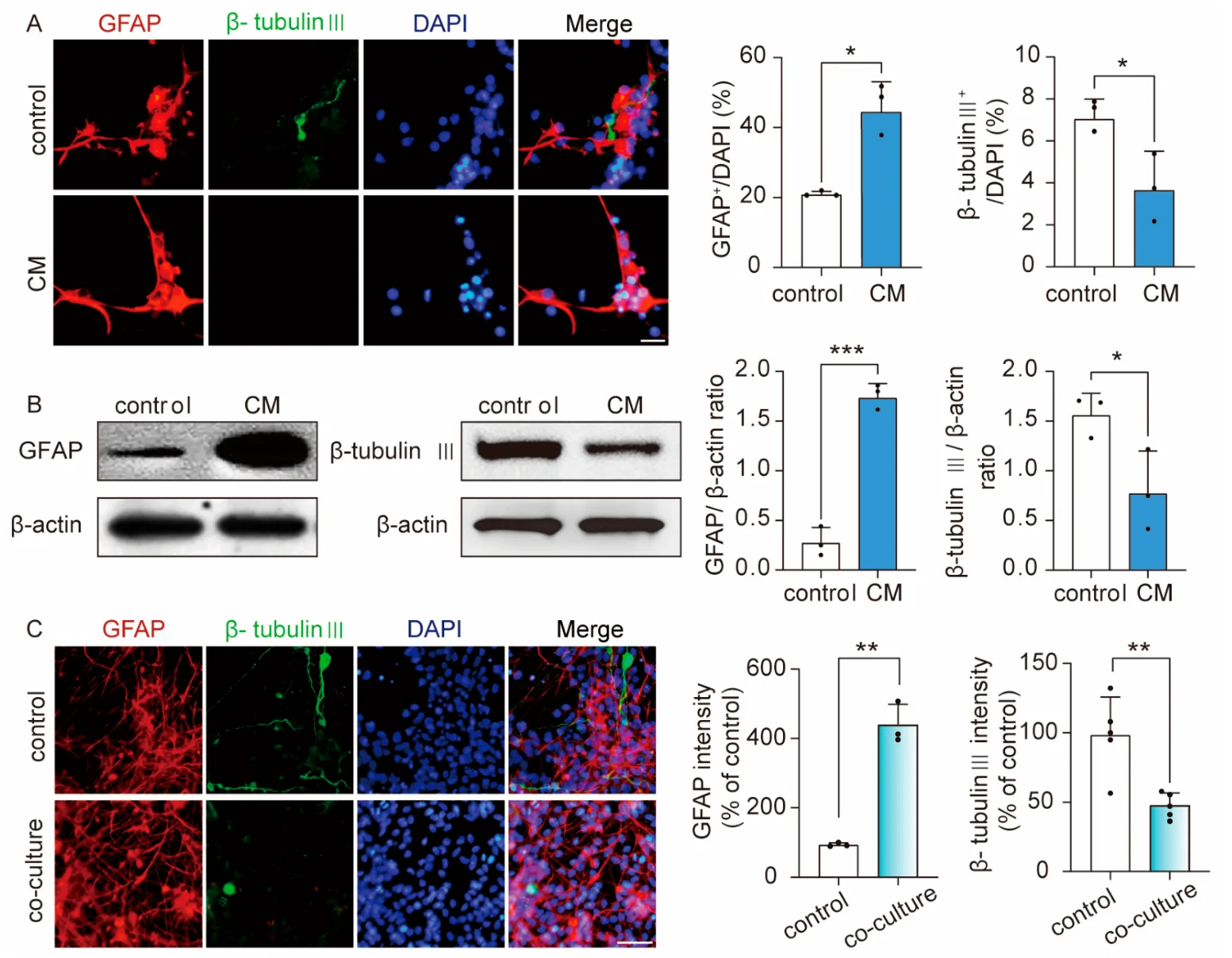
Effects of glial cell niche on NSCs’ differentiation in CM and Transwell co-culture systems. (**A**) Immunostaining and quantification of β-Tubulin III-positive (green) and GFAP-positive (red) cells following CM treatment (*n* = 3), scale bar = 10 μm. (**B**) Western blot analysis of β-Tubulin III and GFAP expression after CM treatment (*n* = 3). (**C**) Immunostaining and quantification of GFAP (red, n = 3) and β-Tubulin III (green, *n* = 5) intensity after culture in the Transwell system, scale bar = 20 μm. Cell nuclei were stained with DAPI (blue). Student’s *t*-test, * *p* < 0.05, ** *p* < 0.01, *** *p* < 0.001.

Both immunostaining and Western blotting were performed to assess NSC differentiation. The results showed that after 7-day culture in CM the proportion of GFAP-positive astrocytes increased, while that of β-tubulin III-positive neurons decreased (Figure 6A,B), suggesting a tendency of NSCs to differentiate into astrocytes rather than neurons. A similar pattern in NSCs’ differentiation tendencies was observed in the Transwell co-culture system (Figure 6C), further confirming the regulatory effect of the glial cell niche on NSCs’ differentiation.

### 2.6. BMP4 Was Involved in the Regulatory Effect of Glial Cell Niche on NSCs

**Figure 7:**
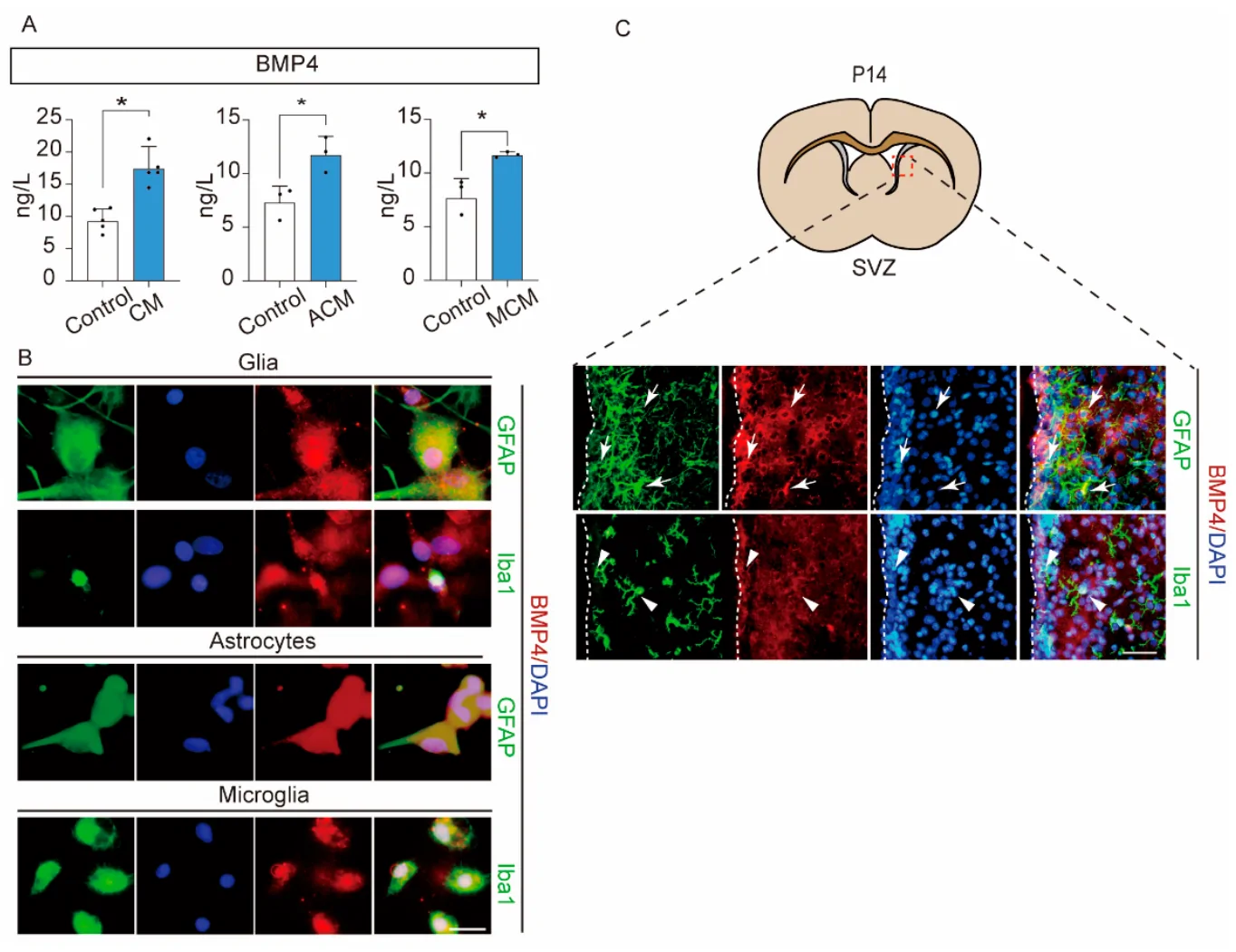
Detection of BMP4 expression in glial cells both in vitro and in vivo. (**A**) Detection of BMP4 in culture supernatants from mixed glial cells (*n* = 5), astrocytes (*n* = 3), and microglia (*n* = 3) using ELISA. (**B**) Immunostaining analysis of BMP4 (red) colocalization with GFAP (green) and Iba1 (green) in cultured cells, scale bar = 10 μm. (**C**) Immunostaining analysis of BMP4 (red) colocalization with GFAP (arrow) and Iba1 (arrowhead) in the SVZ of P14 rat brains (*n* = 3), scale bar = 20 μm. Cell nuclei were stained with DAPI (blue). Student’s *t*-test, * *p* < 0.05.

BMP4 levels in the culture media from mixed glial cells, separately cultured astrocytes, and microglia were quantified by ELISA. Results showed BMP4 levels were significantly elevated (Figure 7A). Immunostaining results further showed the colocalization of BMP4 with GFAP or Iba1 both in cultured cells (Figure 7B) and in brain tissue (Figure 7C).

**Figure 8:**
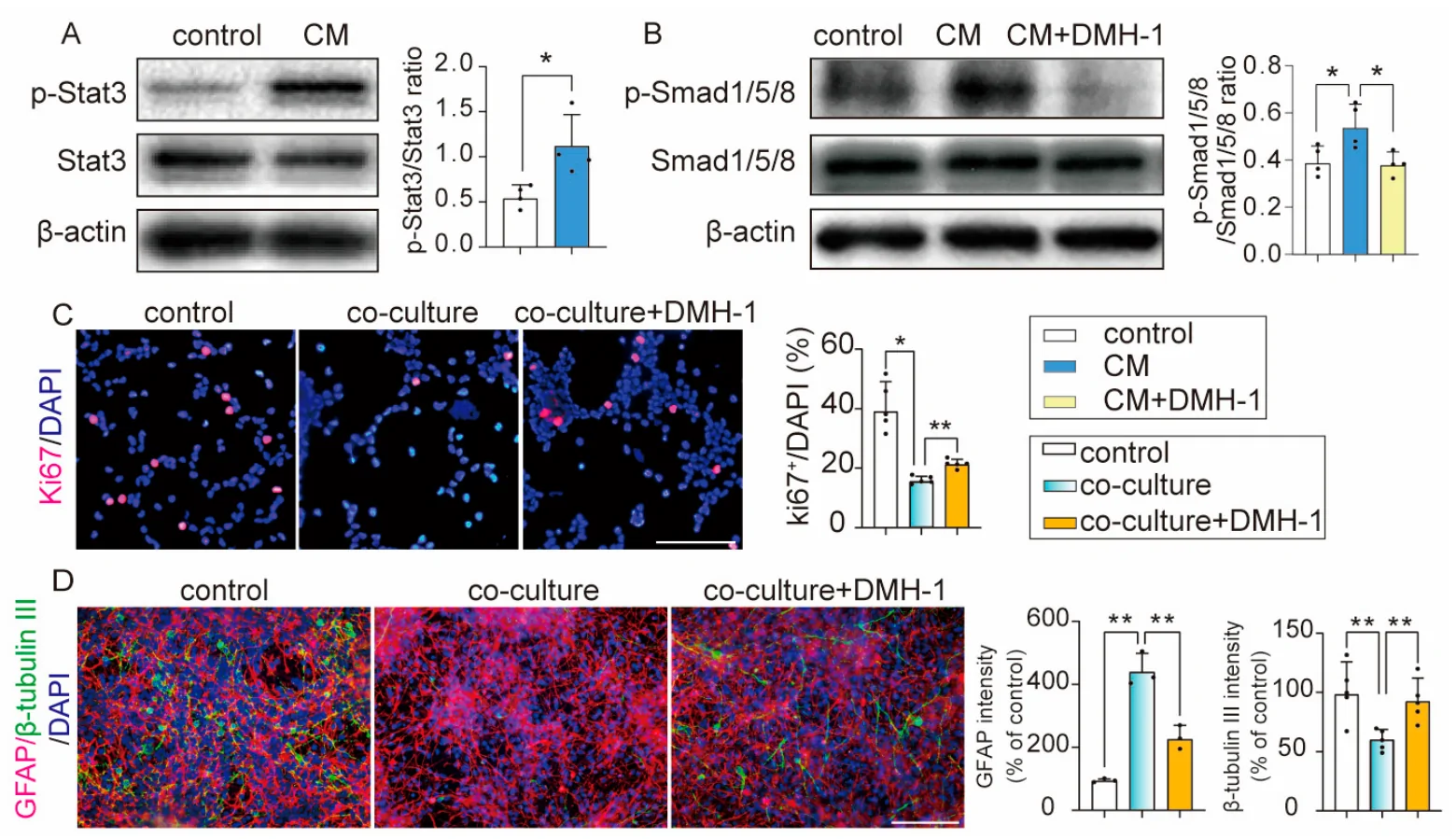
Role of the Smad1/5/8 signaling pathway and Stat3 in regulating NSCs’ behaviors. (**A**,**B**) Western blot analysis of p-Stat3 (*n* = 4) and p-Smad1/5/8 (*n* = 4) in NSCs after being cultured in CM. (**C**) Immunostaining and quantification of Ki67-positive cells (red) after culture in the Transwell system (*n* = 5). (**D**) Immunostaining and quantification of β-Tubulin III-positive (green, *n* = 5) and GFAP-positive (red, *n* = 3) cells after culture in the Transwell system, scale bar = 50 μm. Cell nuclei were stained with DAPI (blue). Data were analyzed using Student’s *t*-test (**A**) or one-way ANOVA followed by LSD (**B**,**D**) or Tamhane’s T2 (**C**) post hoc tests. * *p* < 0.05, ** *p* < 0.01.

As a downstream effector of BMP4 signaling, Smad1/5/8 phosphorylation (p-Smad1/5/8) was detected by Western blotting. Results showed that the levels of p-Smad1/5/8 and p-Stat3 in NSCs were significantly increased after being cultured in CM (Figure 8A,B), indicating the activation of the BMP4–Smad1/5/8 signaling pathway. Furthermore, 20 μM DMH-1, an inhibitor of Smad1/5/8 phosphorylation, was used. Results showed that p-Smad1/5/8 levels were reduced (Figure 8B), accompanied by restored NSC proliferation and reduced astrocytic differentiation (Figure 8C,D).

## 3. Discussion

This study characterized the spatial distribution of microglia, astrocytes and NSCs in the SVZ of early postnatal rats, revealing the cellular composition and interactions of the neurogenic niche. Consistent with previous reports, glial cells exert regulatory effects on NSCs’ activities [10,11]. Our results demonstrated that co-culture with astrocytes and microglia inhibited NSC proliferation, whereas cell viability and astrocytic differentiation of NSCs were promoted. This regulatory effect is associated with glial cell-secreted BMP4 via the BMP4–Smad1/5/8 signaling pathway.

Niche signals in neurogenic regions, the SGZ and SVZ, are crucially required for NSC proliferation and differentiation during neurogenesis [4,5]. Besides the extracellular matrix, cellular components and other physicochemical properties play essential roles in regulating the biological behaviors of NSCs. Our results showed that during the early postnatal development period, astrocytes and microglia were located closely to NSCs in the SVZ. This indicates the possible interaction between glial cells and NSCs during neurogenesis. This result is supported by previous studies that showed the influence of microglia and astrocytes on neurogenesis and gliogenesis [23,24]. The overall role of glial cells in the cellular architecture surrounding NSCs remains insufficiently studied, owing to difficulties in in vitro multicellular culture and complex intercellular interactions in vivo [25,26].

In order to investigate the influence of glial cells on NSCs, we established an in vitro glial cell niche. Glial cells isolated from P1 neonatal rat brains were cultured. The cellular composition of the culture system, consisting of microglia and astrocytes, was similar to that of brain tissue. This indicates the mixed glial cell culture partially reproduced key cellular components of the early postnatal glial niche. Then, NSCs were cultured in the glial cell niche by culturing in glial cell CM or co-culturing with mixed glial cells in Transwell inserts. This model provided an experimental platform for investigating how glial-derived signals and cell–cell interactions influence NSCs’ behaviors in vitro.

CM is a useful approach for determining whether soluble factors released by one cell population under defined conditions can influence recipient cells [27,28]. It represents a one-way interaction model based on pre-collected CM, in which degradation or depletion of secreted factors over time may lead to an underestimation of their effects [29]. In contrast, the Transwell system enables dynamic bidirectional interactions between different cell populations by allowing the continuous exchange of soluble factors through a porous membrane while maintaining physical separation [30]. This model more closely mimics a niche-like microenvironment [29]. These two approaches used in our study are complementary, with the CM reflecting the accumulated glia-derived secreted factors and allowing assessment of BMP4 function within a one-way interaction model, while the Transwell co-culture system captures dynamic bidirectional glia–NSC interactions, thereby more closely recapitulating the physiological niche environment.

Our study showed that NSCs’ viability was enhanced after being cultured in a glial cell niche, consistent with previous findings on individual glial cell types. Evidence indicates that astrocyte-derived extracellular vesicles support NSC survival and function in traumatic brain injury models [31,32]. Signals from glial cells regulate the F-actin dynamics in NSCs through the Smo-Gα-Rho1 signaling axis, thereby promoting NSC reactivation [33]. Similarly, IL-13-pretreated microglia suppress pro-inflammatory responses, thereby indirectly supporting NSC viability [34]. In addition, co-transplantation of astrocytes with NSCs significantly improves NSCs survival, neuronal maturation, and synaptic remodeling [35]. All these data further emphasize the crucial roles of the glial cell niche in regulating NSC viability and function and are also consistent with our results. The CCK-8 result indirectly reflects NSC viability based on the assessment of cellular dehydrogenase activity [36,37], while additional assays are required to specifically assess the effects of CM on NSC survival and apoptosis.

Glial cells regulate NSC proliferation by promoting quiescence or limiting proliferative activity. Whether polarized toward M1 or M2 subtypes, microglia suppress NSC proliferation while enhancing the migration and differentiation of NSCs through inflammatory cytokines and cell–cell contact [38]. Meanwhile, astrocytes activate Id4 expression, which helps maintain NSC quiescence through Notch2 signaling [39]. NKCC1 in glial cells regulated the local ionic environment, affecting NSC membrane potential and osmotic balance to suppress NSC proliferation [40]. Our results showed that the proliferation of NSCs was significantly reduced when cultured in the glial cell niche, consistent with previous studies. These findings suggest that glial cells contribute to the regulation of NSC proliferation within the niche, although further in vivo studies are needed to determine whether these mechanisms are preserved following brain injury.

Our results also showed that in the glial cell niche, the morphological maturation of NSCs was enhanced, as shown by increased process outgrowth [41,42]. Regarding differentiation, the neuronal differentiation of NSCs was inhibited while astrocytic differentiation was promoted. This finding is in line with previous reports showing that microglia promote astrocytic differentiation of NSCs through soluble factor-mediated signaling [38,43]. The existing literature confirms that ferritin secreted by glial niche cells drives iron entry into NSCs, supporting both their self-renewal and differentiation [44]. Combined with reduced proliferation, the increased cell metabolic activity observed after CM treatment may reflect enhanced cellular maturation associated with NSC differentiation, rather than expansion of cell numbers. This finding highlights the potential role of CM in promoting NSCs’ fate transition.

NSC biological behaviors were altered after culture in the glial cell niche using CM or a Transwell co-culture system, indicating that glial-derived soluble factors contribute to NSC regulation [45]. Several studies have confirmed that factors derived from glial cells participate in modulating the local niche, and BMP4 is highly expressed during late embryonic development and early postnatal periods [21,22]. Our results showed that BMP4 was expressed by both astrocytes and microglia isolated from the early postnatal rat cortex. After culture in the glial cell niche, the phosphorylation levels of Smad1/5/8 and Stat3 were significantly elevated, indicating activation of the Smad1/5/8 signaling pathway. The successful restoration of altered NSCs’ biological behavior by DMH-1 application further supports the role of BMP4–Smad1/5/8 signaling in modulating NSCs’ behaviors during early postnatal development.

These findings are consistent with previous studies highlighting the critical role of BMP signaling in NSC fate regulation. BMP4 induces astrocytic differentiation when applied alone but promotes NSC quiescence in the presence of bFGF [46]. BMP4 signaling through Smad1/5/8 has been shown to exert stage-dependent effects during neural development, promoting neurogenesis at early stages and astrocyte generation at later stages [47,48]. In our study, activation of BMP4–Smad1/5/8 signaling following glial niche exposure was associated with reduced NSC proliferation and enhanced astrocytic differentiation. This signaling may contribute to NSC fate regulation by promoting a less proliferative state and activating astroglial-associated programs, potentially involving Stat3 and other astrocyte-promoting pathways. Additional signaling pathways, including Notch and Wnt signaling, as well as factors such as cytokines and extracellular matrix components, may also contribute to NSC fate regulation.

This study has several limitations. Traditional 2D culture systems do not fully replicate the cellular architecture and biomechanical cues of the SVZ, limiting the investigation of long-term glia–NSC interactions. Advanced 3D culture systems may help overcome these limitations and should be explored in future studies. The molecular mechanisms underlying glial–NSC interactions are highly interconnected, forming a complex regulatory network that influences NSC behaviors. Future studies are needed to investigate these combined effects to better understand how glial cells modulate NSC behavior at the molecular level using multi-omics approaches. In addition, while our work reveals mechanisms underlying glial regulation of NSC function, further studies in clinically relevant models are needed to assess their potential for neural repair.

## 4. Materials and Methods

### 4.1. Animals

Pregnant Sprague–Dawley (SD) rats were obtained from the Experimental Animal Center of Xi’an Jiaotong University and kept under standard laboratory conditions: a 12 h light/dark cycle and controlled temperature (22 ± 2 °C) and humidity (60–80%) with free access to water and food. The embryos at E14 were used for NSC culture. The day of delivery was defined as P0. Neonates at P1 were used for primary glial cell culture. Brain tissue was also collected from the offspring on P14. All experimental procedures were conducted in accordance with the Guide for the Care and Use of Laboratory Animals (NIH) and monitored by the Institutional Animal Care and Use Committee (IACUC) of Xi’an Jiaotong University (2021-1835).

### 4.2. Primary Cell Culture

For NSC culture, rat embryos (*n* = 20) were collected at E14 and NSCs were isolated from the cerebral cortex. Following a standardized and laboratory-optimized protocol [49], the cells were cultured in NSC growth medium, comprising a 1:1 mixture of DMEM/F12 (#12400, Gibco, Carlsbad, CA, USA) supplemented with 1% N2 (#17502-048, Gibco, USA), 2% B27 (#17504-044, Gibco, USA), 20 ng/mL EGF (#PHG0311, Gibco, Carlsbad, CA, USA), 10 ng/mL bFGF (#PHG0021, Gibco, Carlsbad, CA, USA) and 1% penicillin–streptomycin (#P1400, Solarbio, Beijing, China). NSCs were passaged every 3–4 days using TrypLE Express (#12604021, Gibco, Carlsbad, CA, USA) and cells in passages 2 and 3 were used in the subsequent experiments. Medium containing DMEM/F12, N2, B27 and 1% fetal bovine serum (FBS, #10270-160, Gibco, Carlsbad, CA, USA) was used for NSC differentiation.

For glial cell culture, P1 rat neonates (*n* = 40) were collected and cells were isolated from the cerebral cortex following a standard protocol [50]. All cells were cultured in glial cell culture medium which contained high-glucose DMEM and 10% FBS, in poly-L-lysine (PLL, #P2100, Solarbio, Beijing, China)-coated T25 flasks. The non-adherent cells were washed away at 30 h after seeding. Then all cells were kept in culture with medium changes every 4 days. Astrocytes and microglia in the primary cultured glial cells were identified by immunofluorescent staining and flow cytometric analysis at the 13th day.

### 4.3. NSC Culture in the Glial Cell Niche

Two approaches were applied to culture NSCs in the glial cell niche. The first approach was culturing NSCs in glial cell-conditioned medium (CM). To obtain the glial cell CM, mixed glial cells were maintained in glial cell culture medium for 13 days followed by incubation in NSC growth medium for an additional 24 h. The supernatant was then collected and used as CM. The second way was co-culturing NSCs with mixed glial cells via a Transwell (0.4 μm, #3470, Corning Incorporated, Corning, NY, USA) system. NSCs were cultured in the lower chamber and mixed glial cells were seeded on the membrane of the insert. The co-culture medium was NSC growth medium and was refreshed every 2–3 days.

For pharmacological inhibition experiments, 20 μM DMH-1 (#T1942, TargetMol, Boston, MA, USA), an inhibitor of Smad1/5/8 phosphorylation, was added to the culture medium during CM treatment or Transwell co-culture.

### 4.4. Cell Viability Assay

Cell viability was assessed using the Cell Counting Kit-8 (CCK-8, #CK04, Dojindo Laboratories, Kumamoto, Japan). Absorbance at 450 nm was recorded using an Epoch microplate reader (BioTek, Winooski, VT, USA). NSCs were seeded at 3 × 10^4^ cells per well in PLL-precoated 96-well plates and maintained in NSC growth medium for 24 h. Then, the medium was replaced with CM in different concentrations. Accordingly, the cultured NSCs were divided into five groups: control (NSC growth medium), 1/8 CM (the volume ratio of CM to NSC culture medium), 1/4 CM, 1/2 CM and undiluted CM.

### 4.5. Live-Cell Imaging

Live-cell imaging was used for cell growth analysis. NSCs were seeded in 96-well plates, and live-cell images were captured. Total cell number and cell-covered area were quantified by a Cytation 5 imaging plate reader (BioTek, Winooski, VT, USA) at different time points during culture.

### 4.6. Flow Cytometry Analysis

Flow cytometry analysis was used to identify the proportion of astrocytes and microglia in the primary cultured mixed glial cells and for NSC cycle analysis after culture in CM. Cultured glial cells were stained with 5 μL CD11b-APC (#562102, BD Biosciences, Franklin Lakes, NJ, USA) and 1 μL GFAP-Alexa Fluor 488 (#53989280, Thermo Fisher Scientific, Waltham, MA, USA) for 30 min after fixation and permeabilization (#554714, BD Biosciences, Franklin Lakes, NJ, USA). Staining and analyzing were performed according to the instructions. For cell cycle analysis, cultured NSCs were fixed in 75% ethanol at 4 °C overnight and labeled with propidium iodide (#P4170, Sigma, Darmstadt, Germany) before assessing [51]. Flow cytometry was used to determine the percentages of cells in the G0/G1, S, and G2/M phases.

### 4.7. Morphological Investigation and Immunofluorescence Staining

The morphological features of NSCs in 6-well plates (5 × 10^5^ cells/well) were observed using a light microscope and analyzed using ImageJ 1.54f software after culture in CM for 24 h. Immunofluorescence staining was performed both on brain slices and cultured cells according to the standard protocol and optimized in our lab [49,52]. Briefly, samples were fixed with 4% paraformaldehyde (PFA, #P1117, Solarbio, Beijing, China). Bovine serum albumin (BSA, #A9418, Darmstadt, Germany) was used for blocking. Primary antibodies, including mouse anti-nestin (1:500, #NBP1-92717, Novus Biologicals, Centennial, CO, USA), rabbit anti-GFAP (1:500, NB-300-141, Novus Biologicals, Centennial, CO, USA), mouse anti-GFAP (1:500, #3670, Cell Signaling Technology, Danvers, MA, USA), rabbit anti-Iba1 (1:500, #019-19741, Wako, Osaka, Japan), and mouse anti-β-tubulin III (1:500, #ab78078, Abcam, Cambridge, UK), were used for cell type assessment, and rabbit anti-Ki67 (1:200, #9129, Millipore, Billerica, MA, USA) and mouse anti-BMP4 (1:100, #sc-12721, Santa Cruz Biotechnology, Dallas, TX, USA) were used to assess cellular behaviors and signaling pathways. Secondary antibodies, including Alexa Fluor 488-conjugated goat anti-rabbit IgG (#A-11008, Thermo Fisher Scientific, Waltham, MA, USA) and donkey anti-mouse IgG (#A-21202, Thermo Fisher Scientific, Waltham, MA, USA) and Alexa Fluor 594-conjugated donkey anti-rabbit IgG (#A-21207, Thermo Fisher Scientific, Waltham, MA, USA) and goat anti-mouse IgG (#A-11005, Abcam, Cambridge, UK) were used. Cell nuclei were stained with DAPI (#S2110, Solarbio, Beijing, China) and imaging of slices was performed using a fluorescence microscope (BX57, Olympus Corporation, Tokyo, Japan). Photographs were then processed and analyzed via ImageJ software. The positively stained cells were quantified on three brain or cell slices with 5 random fields from each slice.

### 4.8. Western Blotting

Western blotting analysis was performed to detect GFAP and β-tubulin expression to evaluate NSC differentiation and also to detect p-Stat3 and p-Smad1/5/8 after culture in CM for 4 h to explore the related signaling pathways. NSCs were seeded into PLL-coated 6-well plates at a density of 6 × 10^5^ cells/well and Western blotting was performed as previously described [49]. Briefly, cells were lysed in RIPA buffer (#89900, Thermo Fisher Scientific, Waltham, MA, USA) supplemented with protease and phosphatase inhibitors (#4693159001 and 04906845001, Roche, Basel, Switzerland). Cell lysates were incubated on ice for 30 min, centrifuged at 12,000 rpm for 15 min at 4 °C, mixed with 5× SDS-PAGE loading buffer, and boiled at 95 °C for 5 min. Equal amounts of protein (20 μg per lane) were separated by 10% SDS-PAGE and transferred onto 0.22 μm PVDF membranes (Millipore, Billerica, MA, USA). After blocking with 5% nonfat milk dissolved in TBS-T buffer (1×TBS containing 0.1% Tween-20) for 2 h at room temperature, membranes were incubated with primary antibodies diluted in Universal Antibody Diluent (#WB500D, NCM Biotech, Suzhou, China) overnight at 4 °C. The primary antibodies used included mouse anti-GFAP (1:1000, #3670, Cell Signaling Technology, Danvers, MA, USA), mouse anti-β-tubulin III (1:1000, #ab78078, Abcam, UK), rabbit anti-Stat3 (1:1000, #12640, Cell Signaling Technology, Danvers, MA, USA), rabbit anti-p-Stat3 (1:1000, #9145, Cell Signaling Technology, Danvers, MA, USA), rabbit anti-p-Smad1/5/8 (1:800, #D5B10, Cell Signaling Technology, Danvers, MA, USA), rabbit anti-Smad1/5/8 (1:200, #sc6031R, Santa Cruz Biotechnology, Dallas, TX, USA) and mouse anti-β-actin (1:5000, 60008-1-Ig, Immunoway, Wuhan, China). After washing with TBS-T buffer, the membranes were incubated with HRP-conjugated secondary antibodies diluted in the same antibody diluent for 2 h at room temperature. Secondary antibodies, including HRP-conjugated goat anti-mouse IgG (1:5000, SA00001-1, Immunoway, Wuhan, China) and HRP-conjugated goat anti-rabbit IgG (1:5000, SA00001-2, Immunoway, Wuhan, China), were used. All bands were visualized using an ECL kit (#WBKLS0500, Millipore, Billerica, MA, USA) and the images were acquired using X-ray imaging or a luminescence imaging system. Protein band intensity was normalized to β-actin and analyzed using ImageJ software.

### 4.9. Quantification of BMP4 by ELISA

A quantitative ELISA kit (#MM-0207R1, Meimian, Yancheng, China) was used to measure BMP4 levels in the culture media collected from mixed glial cell, astrocyte, or microglial culture. Following the instructions, culture medium (10 μL) from different cultures was collected and incubated with the standard substance (50 μL) at 37 °C for 30 min. After thoroughly washing, the enzyme labeling reagent (50 μL) was added. Subsequently, chromogenic reagents were added and incubation was performed at 37 °C in the dark for 10 min. Absorbance values at 450 nm were measured using the microplate reader within 15 min, and BMP4 concentrations were calculated based on the standard curve.

### 4.10. Statistical Analyses

Data were presented as the mean ± SD and all data were analyzed using SPSS 18.0 and GraphPad Prism 8.0 software. Data distribution was assessed for normality using the Shapiro–Wilk test, and homogeneity of variances was evaluated using Levene’s test. Statistical analysis was performed with unpaired Student’s *t*-test for two groups or one-way ANOVA followed by LSD test (equal variances) or Tamhane’s T2 test (unequal variances) for multiple comparisons. Two-way ANOVA followed by Sidak’s post hoc test was used for analyses involving two independent variables. A *p* value < 0.05 was considered statistically significant.

## 5. Conclusions

In this study, we observed the spatial distribution of microglia, astrocytes, and NSCs in the rat SVZ during early postnatal development, providing insights into the cellular components of the NSC niche and the potential cellular interactions. We further found that, under our in vitro glial cell co-culture models, NSC proliferation and neuronal differentiation were suppressed, while cell viability and astrocytic differentiation were enhanced by both CM treatment and Transwell co-culture. BMP4 secreted by glial cells was involved in regulating NSCs’ biological behaviors, potentially through the BMP4–Smad1/5/8 signaling pathway. Taken together, our findings provide insights into the glial regulation of NSC fate through BMP4-mediated signaling and may support future studies on neurogenesis and regeneration.

## Data Availability

The original contributions presented in this study are included in the article. Further inquiries can be directed to the corresponding author.

## Abbreviations

The following abbreviations are used in this manuscript:

BSA	bovine serum albumin
BMP	bone morphogenetic protein
bFGF	basic fibroblast growth factor
CCK-8	cell counting Kit-8
CM	conditioned medium
DAPI	4’,6-diamidino-2-phenylindole
DMH-1	dorsomorphin homolog 1
DMEM/F12	Dulbecco’s modified Eagle medium/nutrient mixture F-12
ECL	enhanced chemiluminescence
EGF	epidermal growth factor
ELISA	enzyme-linked immunosorbent assay
FBS	fetal bovine serum
GFAP	glial fibrillary acidic protein
HRP	horseradish peroxidase
Iba1	ionized calcium binding adaptor molecule 1
Id4	inhibitor of DNA binding 4
IgG	immunoglobulin G
IL-13	interleukin-13
NKCC1	Na^+^-K^+^-2Cl^−^ cotransporter 1
NSCs	neural stem cells
PI	propidium iodide
PLL	poly-L-lysine
PFA	paraformaldehyde
PVDF	polyvinylidene fluoride
SGZ	subgranular zone
Stat	signal transducers and activators of transcription
SVZ	subventricular zone
